# Expansion of the Multi-Link Frontier™ Coronary Bifurcation Stent: Micro-Computed Tomographic Assessment in Human Autopsy and Porcine Heart Samples

**DOI:** 10.1371/journal.pone.0021778

**Published:** 2011-07-21

**Authors:** Stefan Kralev, Benjamin Haag, Jens Spannenberger, Siegfried Lang, Marc A. Brockmann, Soenke Bartling, Alexander Marx, Karl-Konstantin Haase, Martin Borggrefe, Tim Süselbeck

**Affiliations:** 1 I. Department of Medicine, Faculty of Medicine Mannheim, University of Heidelberg, Mannheim, Germany; 2 Department of Neuroradiology, Faculty of Medicine Mannheim, University of Heidelberg, Mannheim, Germany; 3 Department of Clinical Radiology and Nuclear Medicine, Faculty of Medicine Mannheim, University of Heidelberg, Mannheim, Germany; 4 Department of Clinical Pathology, Faculty of Medicine Mannheim, University of Heidelberg, Mannheim, Germany; 5 II. Department of Internal Medicine, Hospital Steinberg, Reutlingen, Germany; University of Modena and Reggio Emilia, Italy

## Abstract

**Background:**

Treatment of coronary bifurcation lesions remains challenging, beyond the introduction of drug eluting stents. Dedicated stent systems are available to improve the technical approach to the treatment of these lesions. However dedicated stent systems have so far not reduced the incidence of stent restenosis. The aim of this study was to assess the expansion of the Multi-Link (ML) Frontier™ stent in human and porcine coronary arteries to provide the cardiologist with useful in-vitro information for stent implantation and selection.

**Methodology/Principal Findings:**

Nine ML Frontier™ stents were implanted in seven human autopsy heart samples with known coronary artery disease and five ML Frontier™ stents were implanted in five porcine hearts. Proximal, distal and side branch diameters (PD, DD, SBD, respectively), corresponding opening areas (PA, DA, SBA) and the mean stent length (L) were assessed by micro-computed tomography (micro-CT). PD and PA were significantly smaller in human autopsy heart samples than in porcine heart samples (3.54±0.47 mm vs. 4.04±0.22 mm, p = 0.048; 10.00±2.42 mm^2^ vs. 12.84±1.38 mm^2^, p = 0.034, respectively) and than those given by the manufacturer (3.54±0.47 mm vs. 4.03 mm, p = 0.014). L was smaller in human autopsy heart samples than in porcine heart samples, although data did not reach significance (16.66±1.30 mm vs. 17.30±0.51 mm, p = 0.32), and significantly smaller than that given by the manufacturer (16.66±1.30 mm vs. 18 mm, p = 0.015).

**Conclusions/Significance:**

Micro-CT is a feasible tool for exact surveying of dedicated stent systems and could make a contribution to the development of these devices. The proximal diameter and proximal area of the stent system were considerably smaller in human autopsy heart samples than in porcine heart samples and than those given by the manufacturer. Special consideration should be given to the stent deployment procedure (and to the follow-up) of dedicated stent systems, considering final intravascular ultrasound or optical coherence tomography to visualize (and if necessary optimize) stent expansion.

## Introduction

The presence of a bifurcation lesion in patients with coronary artery disease (CAD) is a frequent morphology (8.5%). This incidence was found by a recent study investigating a cohort of 6129 consecutive patients undergoing coronary stent implantation [1]. In most lesion types, drug-eluting stents (DES) have reduced the incidence of revascularization after percutaneous coronary intervention (PCI [2], [3]), but in the case of bifurcation lesions, rates for adverse clinical events still remain high. A recent study investigating bifurcation stenting with sirolimus eluting stents reported in-stent restenosis rates in main vessel and/or side branch of between 6.6% (culotte technique) and 12.1% (crush technique [4]). Before that, main vessel and side branch restenosis rates of >25% were reported [5]–[7], with side branch restenosis considered as a main problem [8].

The existence of an ostial and bifurcation lesion itself is – besides long stents, overlapping stents, suboptimal stent results, stenting of small vessels, existence of multiple lesions, etc – a predictor for late stent thrombosis and restenosis [9], [10]. The high rates of restenosis were one of the reasons for the development of dedicated stent systems (DSS) but according to current guidelines for PCI [11], so far there is no recommendation for the use of DSS. Angiographic success, clinical outcome and development of DSS for coronary bifurcation lesions still remain a challenge and the European Bifurcation Club concluded in a recent state-of-the-art paper that the role of DSS is still the subject of debate, awaiting the “magic” device [12]. Micro-CT has been utilized to investigate oversized postdilation of DES [13], but no micro-CT data are available regarding DSS.

The aim of this study was to assess the expansion of the DSS Multi-Link (ML) Frontier™ (Abbott Vascular, Santa Clara, CA, USA) in human and porcine coronary arteries to provide the cardiologist with useful in-vitro information for stent implantation and selection, contributing to the development of these devices and the treatment of coronary bifurcation lesions.

## Methods

The study was performed in accordance with federal laws and regulations, international accreditation standards, institutional policies and the local ethics committee (Medical Ethic Commission II, Faculty of Medicine Mannheim, University of Heidelberg). Written informed consent was obtained from all patients by the Department of Anatomy, University of Heidelberg, and data were analyzed anonymously.

### Investigation background

**Figure 1:**
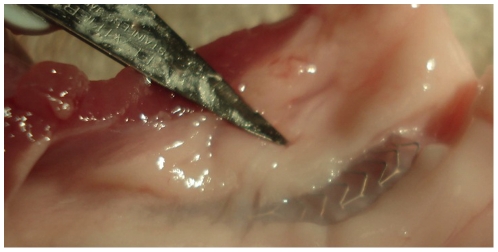
ML Frontier™ stent in porcine coronary artery. Immediately after excisement the ML Frontier™ stents were implanted in fresh vascular bifurcation tissue in the porcine heart samples.

In this *in vitro* study, seven autopsy heart samples from human subjects with known CAD and five porcine heart samples were appropriated. Human and porcine heart samples were immediately preserved with static cold flush with crystalloid solution [14]. Nine stents were implanted in human non-stenotic bifurcations 24–48 h after explantation and five stents were implanted in fresh vascular porcine bifurcation tissue 24 h after excisement (Figure 1). All heart samples were routinely processed for cardiac catheterization with standard 5, 6 or 7F guiding catheters and 0.014-inch floppy guide wires. Because of anonymization no detailed information about the CAD status was available.

### Stent implantation

In total, 14 ML Frontier™ stents with a nominal size of 3.0 mm×18 mm were successfully implanted in appropriate bifurcations under fluoroscopic control. Coronary intervention was performed each time using high-pressure stent deployment as recommended by the manufacturer (14 atm, duration 30–35 s). Angiography was then performed and the coronary perfusion was graded according to the Thrombolysis in Myocardial Trial (TIMI) classification [15]. The proximal and distal reference luminal diameters of the main vessels and the side branches were assessed before stent implantation by quantitative coronary angiography (QCA) and the stent/artery ratios were calculated.

### Micro-CT

**Figure 2:**
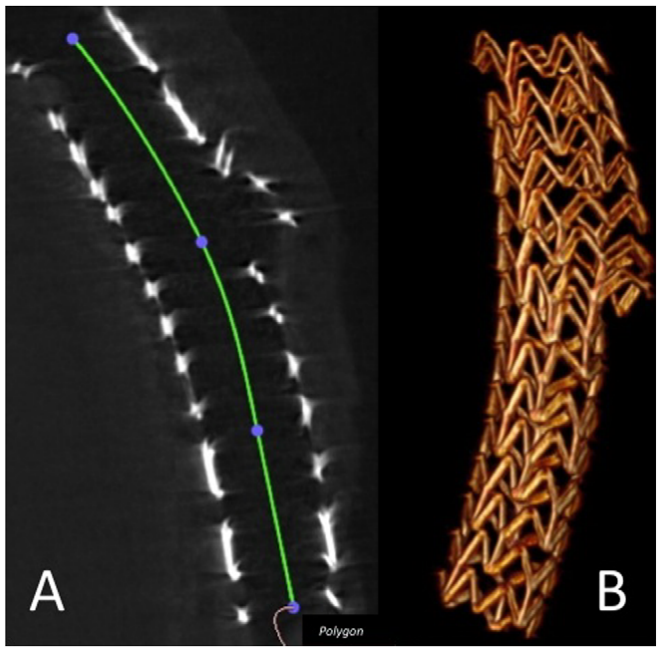
Assessment of the stent length. 2D measurement (panel A) and 3D visualization (panel B) of stent length by micro-CT.

**Figure 3:**
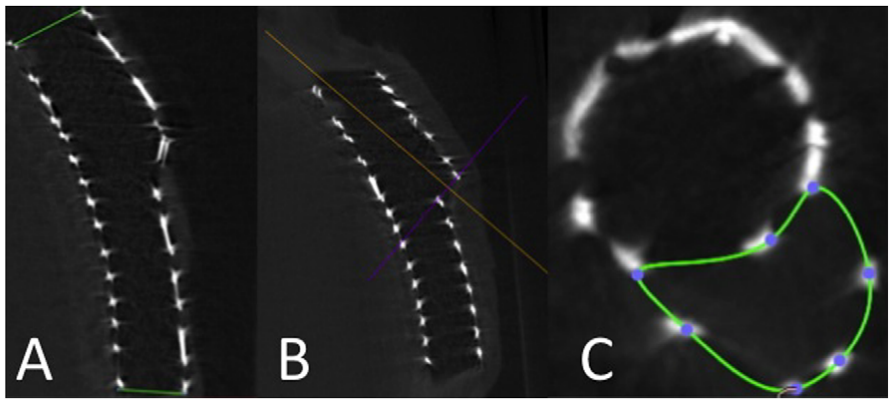
Assessment of the stent opening diameters and areas. Diameters were measured by aligning the z-axis with the centreline of the stent looking exactly perpendicular into the stent lumen (panel A, panel B). Panel C depicts the measurement of the side branch opening area (SBA). Measurements were performed in multiplanar reformation (MPR) mode.

The stented segments were each prepared in a conical 50 ml polypropylene tube (BD Falcon™ Tube, Becton Dickinson, Franklin Lakes). Micro-CT was performed using an industrial micro-CT system (Yxlon Y.Fox, Yxlon International GmbH, Hamburg, Germany). A total of 1200 projections were acquired within 40 s of scan time (2×2 binning of the detector; 944×704 pixel; 360° rotation; 90 µA; 160 kV). Proprietary scanner control software was used to monitor the scanning process, and to save projection data. Standard cone-beam CT reconstruction was performed using a filtered back-projection algorithm (Reconstruction Studio Version 1.2, Comet GmbH, Garbsen, Germany) with Shepp-Logan filter applied. The reconstructed volumes (512 Matrix) were reviewed in multiplanar reformation (MPR) as well as in a 3D volume rendering mode using OsiriX-software (v3.5.1). Data sets were reviewed in MPR (OsiriX) and 3D volume rendering using standard radiological equipment (I-View 3D 1.0.2.3, TeraRecon Inc., San Mateo, USA). After that, devices were surveyed and proximal and distal stent diameters (PD, DD), side branch diameters (SBD), mean stent length (L), as well as the corresponding opening areas (PA, DA, SBA) were measured. The measurements were carried out in MPR-mode. The stent length (Figure 2, panel A), stent diameters and sidebranch diameters (Figure 3, panel A and B) were measured aligning the z-axis with the centreline of the stent looking exactly perpendicular into the stent lumen. In the real world stent opening is not always exactly circular, consequently the stent expansion cannot always be assessed by measuring a diameter, and two orthogonal angiographic views or IVUS (intravascular ultrasound, visualizing vessel lumen and stent opening area) are recommended for evaluation of stent expansion. As with IVUS, micro-CT also offers the possibility of visualizing the stent opening area. Therefore, in this study, in all cases both the diameters and the opening areas were measured and compared with the data given by the manufacturer. The compliance chart of the manufacturer contains the diameters of the stent, so the corresponding opening areas were calculated by applying the circle area formula Area = πr^2^ with r describing the radius of the circle, conforming to 1/2 the diameter. All opening areas were first manually traced by the observer and then determined by the software (Figure 3, panel C, sidebranch opening area).

### Statistical analysis

Deviations from values given by the manufacturer were evaluated by the one sample t test. Data are presented as mean ± standard deviation (SD). Values of p<0.05 (two-tailed) were considered statistically significant. The calculations were performed using SPSS-Software (SPSS-Software GmbH, München, Germany).

## Results

**Figure 4:**
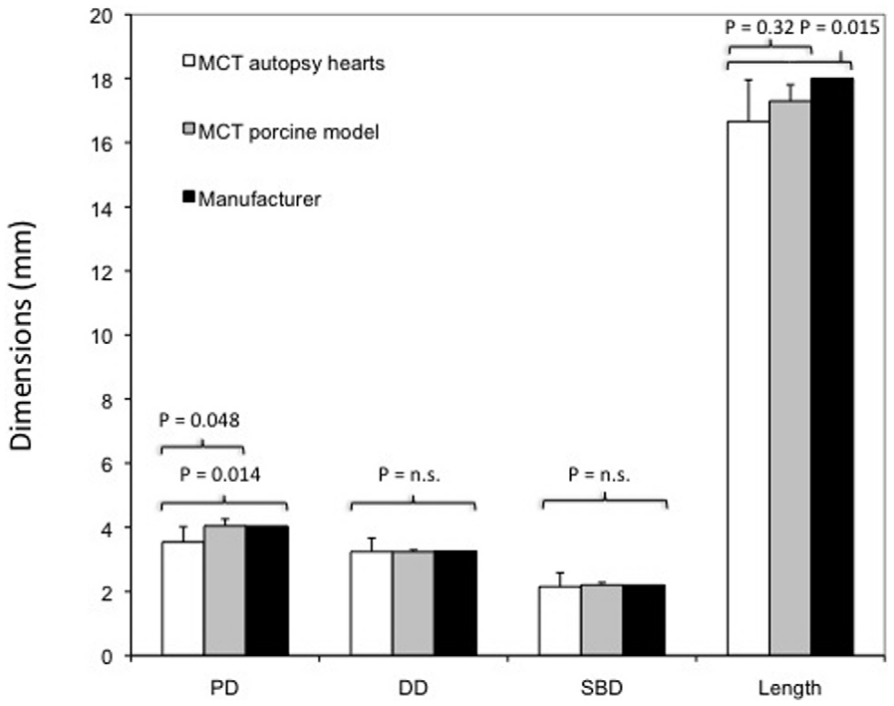
Comparison of stent dimensions between autopsy hearts, porcine model and data given by the manufacturer. Proximal stent diameters (PD) were significantly smaller in human autopsy heart samples than in porcine heart samples (P = 0.048) and than those given by the manufacturer (P = 0.014). The mean stent length was smaller in human autopsy hearts than in porcine heart samples (P = 0.32), and also significantly smaller than that given by the manufacturer (P = 0.015). Side branch diameters (SBD) and distal stent diameters (DD) did not differ significantly.

All DSS showed a successful angiographic result with a TIMI 3 flow. Detailed measurements of opening areas, corresponding diameters, stent lengths, reference luminal diameters and stent-to-artery ratios are presented in Table 1. The proximal stent diameters assessed in human autopsy heart samples were significantly smaller than in porcine heart samples (PD: 3.54±0.47 mm human vs. 4.04±0.22 mm porcine, p = 0.048) and than those given by the manufacturer (PD: 3.54±0.47 mm human vs. 4.03 mm manufacturer, p = 0.014). The mean stent length assessed in human autopsy heart samples was smaller than in porcine heart samples (L: 16.66±1.30 mm human vs. 17.30±0.51 mm porcine, p = 0.32) and significantly smaller than that given by the manufacturer (L: 16.66±1.30 mm human vs. 18 mm manufacturer, p = 0.015). Measured values of the distal stent diameter (DD: 3.24±0.41 mm human vs. 3.26 mm porcine, p = 0.91) and side branch diameter (SBD: 2.15±0.44 mm human vs. 2.2 mm porcine, p = 0.75) showed no significant difference between human autopsy heart samples, porcine heart samples and data given by the manufacturer (Figure 4).

**Table 1:** Micro-CT analysis of stent diameters, opening areas and stent length.

Heart/Stent^n^	Stent diameter/opening area (mm/mm^2^)			Stent length
*Human autopsy hearts*	Proximal	Distal	Bifurcation strut	
I/1^1^	3.98/12.44	3.86/11.70	2.54/5.07	15.8
II/2^2^	2.45/4.70	2.55/5.12	1.47/1.69	14.2
II/3^4^	3.30/8.55	3.30/8.55	2.22/3.87	15.5
III/4^3^	3.50/9.62	2.90/6.61	1.50/1.77	17.6
III/5^4^	3.44/9.29	3.53/9.79	1.99/3.11	16.1
IV/6^4^	3.70/10.75	3.42/9.19	2.61/5.35	17.5
V/7^2^	3.67/10.58	2.81/6.20	2.42/4.59	17.8
VI/8^1^	4.04/12.82	3.49/9.57	2.06/3.33	17.9
VII/9^1^	3.78/11.22	3.34/8.76	2.55/5.11	17.5
RLD*	3.73±0.28	3.17±0.15	1.79±0.30	
*Stent/artery ratio* †	1.08 ∶ 1	1.03 ∶ 1	1.23 ∶ 1	
*Stent/artery ratio* ‡	0.95 ∶ 1	1.02 ∶ 1	1.20 ∶ 1	
***Porcine heart model***				
I/10^1^	4.23/14.05	3.24/8.23	2.28/4.07	18.0
II/11^2^	3.86/11.70	3.19/8.01	2.29/4.12	17.4
III/12^4^	3.79/11.31	3.33/8.72	2.12/3.53	17.2
IV/13^3^	4.03/12.75	3.19/8.02	2.17/3.71	17.3
V/14^3^	4.29/14.41	3.25/8.32	2.16/3.65	16.6
RLD*	2.92±0.19	2.31±0.17	1.48±0.33	
*Stent/artery ratio* †	1.38 ∶ 1	1.41 ∶ 1	1.49 ∶ 1	
*Stent/artery ratio* ‡	1.00 ∶ 1	0.99 ∶ 1	1.00 ∶ 1	
***Manufacturer*** **	*4.03/12.76*	*3.26/8.35*	*2.20/3.80*	*18.0*

n = Location of bifurcation:

1 left anterior descending (LAD)/ramus diagonalis (RD) I,

2 LAD/RD II,

3 LAD/left circumflex,

4 right coronary artery: ramus interventricularis posterior/ramus posterolateralis dexter.

* Measured before stent implantation by QCA.

† Calculated with stent diameter given by manufacturer.

‡ Calculated with stent diameter assessed by Micro-CT.

** Opening area was calculated using the circle formula area = π radius^2^. RLD: reference luminal diameter; QCA: quantitative coronary angiography.

**Figure 5:**
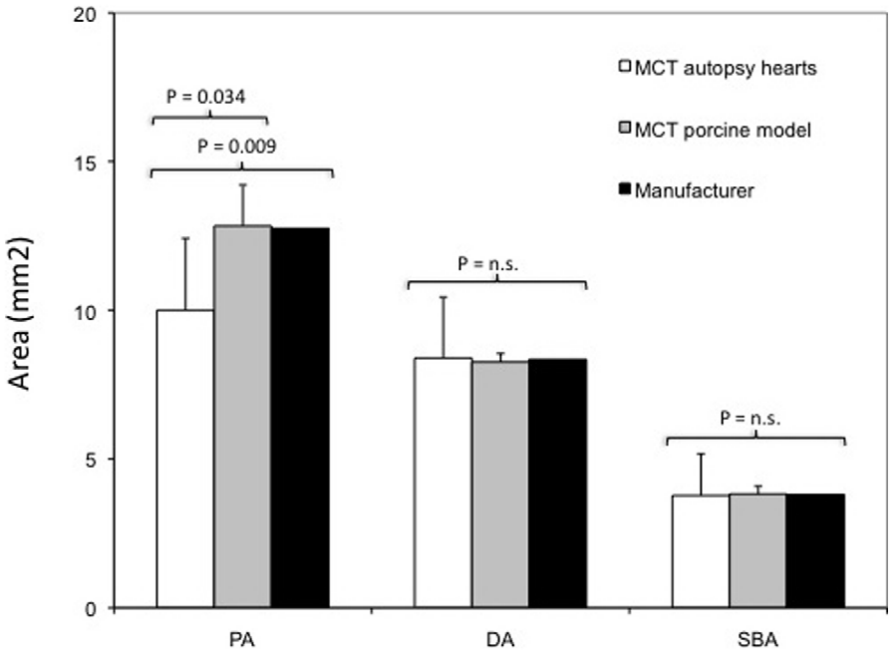
Comparison of stent areas between autopsy hearts, porcine model and data given by the manufacturer. Proximal stent opening areas (PA) were significantly smaller in autopsy human heart samples than in porcine heart samples and than those given by the manufacturer. Side branch opening areas (SBA) and distal stent opening areas (DA) did not differ significantly.

Correspondingly, the proximal stent opening area was significantly smaller than the area measured in porcine heart samples (PA: 10.00±2.42 mm^2^ human vs. 12.84±1.38 mm^2^ porcine, p = 0.034) and the area given by the manufacturer (PA: 10.00±2.42 mm^2^ human vs. 12.76 mm^2^ manufacturer, p = 0.009, calculated with the proximal diameter of the compliance chart). The distal stent opening area (DA: 8.39±2.06 mm^2^ human vs. 8.35 mm^2^ porcine, p = 0.96) and the side branch opening area (SBA: 3.77±1.40 mm^2^ human vs. 3.80 mm^2^ porcine, p = 0.94) did not differ significantly from areas measured in porcine heart samples or data given by the manufacturer (Figure 5).

**Figure 6:**
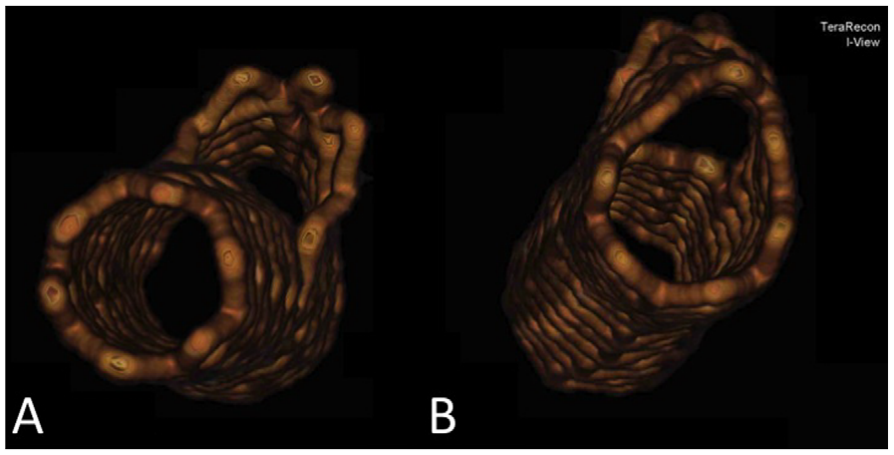
3D Visualization of the ML Frontier™ stent after expansion. Adequate expansion of distal opening (panel A) and inadequate expansion of the proximal stent opening (panel B).

Exact 2D measurement and 3D visualization of the stent length is depicted in Figure 2, panels A and B. Adequate expansion of the distal opening and the side branch opening is depicted in Figure 6, panel A; inadequate expansion of the proximal opening is shown in Figure 6, panel B.

## Discussion

Micro-CT is an independent method capable of measuring stent cells with high precision [13], [16]. This is the first study that has investigated a DSS by micro-CT, visualizing the deployment behaviour and assessing expansion in different tissue models. Proximal diameters of the devices implanted in human autopsy hearts were significantly smaller than in porcine heart samples and than the predicted diameter from the manufacturer's compliance chart. In comparison to the porcine heart samples, as in real world stenting conditions, the human heart samples had atherosclerosis. Atherosclerosis is associated with a higher vessel wall resistance and this might have led to a suboptimal expansion of the stent in comparison to the porcine heart samples and in comparison with the data given by the compliance chart. Proximal vessel diameters are generally larger than distal vessel diameters, which should ideally also be factored in the stent-balloon design. But conical ballons (proximal balloon ending larger than distal ballon ending) are not available, so this might be a reason why suboptimal stent expansion was observed mainly in the proximal stent part. Similar, it must be taken in account that coronary arteries are not linear so that vessel wall resistance in the curved part and/or shear forces of the coronary arteries due to atherosclerosis might hinder complete length expansion. Our results reflect findings of a previous study in which intravascular ultrasound (IVUS) was used to investigate four different stents demonstrating that the minimal stent diameters measured by IVUS were significantly smaller than those predicted by the manufacturers [17]. Regarding clinical data relating to the ML Frontier™, the FRONTIER stent registry [18] reported an initial device success of 91%. At 6 month follow-up, the MACE rate was 17.1% with a main branch in-stent restenosis (ISR) of 25.3%. Newer data evaluating the ML Frontier™ [19] reported a device success of 95% (postprocedural main vessel stenosis 19%) with a follow-up ISR rate of 29% (in-stent late lumen loss 0.52±0.44 mm). The results of the present study might contribute to a better understanding of these clinical trials, suggesting that in human heart samples with CAD the proximal part of the ML Frontier™ shows a smaller expansion characteristic than in porcine heart samples or in a water bath.

Our results are in accordance with the results of another study, reporting an incomplete apposition also at the proximal edge of the DSS AXXESS in two of 139 cases [20]. Although there is a case report of the ML Frontier™, showing an adequate proximal stent apposition documented by IVUS [21]. On balance, we agree with Bhalla *et al.*, recommending careful stent deployment [22] and propose special consideration of balloon inflation pressure and duration, postdilation of the proximal stent part and final kissing balloon technique, respecting the individual lesion type of the patient to avoid stent malapposition.

Stent malapposition and its pathophysiological role in both ISR and stent thrombosis is of major importance. One of the leading sources of ISR is local vascular healing response associated with intimal hyperplasia [23]. Main predictors of ISR are final minimal luminal diameter, stent length and structural deformation of the stents with suboptimal vessel wall coverage [24], [25]. Insufficiently enlarged stent cells may also cause thrombus formation [26], [27]. Several important studies have demonstrated that stent underexpansion/malapposition is one of the major reasons for stent thrombosis [26], [28]–[31]. Focusing on data for bifurcation stenting, fewer data are available but similar mechanisms are thought to be implicated. Good positioning of the stent struts against the vessel wall, adequate stent openings and the stent length are – in addition to other factors such as delta angle, etc – crucial factors for successful bifurcation stenting [27], [32]–[34]. The results of the present study suggest that in human autopsy hearts with CAD the stent length might be slightly smaller than expected. More micro-CT studies with newer stent devices are needed to clarify this point. We agree with Mortier e*t al.* who recommend careful selection of stent devices [16], and want to draw special attention also to the stent length. Therefore, after complex PCI with heavy calcification, need for multiple balloon inflation, etc, critical observation of the angiographic result in two orthogonal perspectives should be performed. In some cases IVUS, optical coherence tomography (OCT) or X-ray visual enhancement [35] might provide additional useful information, enabling optimization of the stent deployment.

Another conclusion of this study is that micro-CT is a feasible tool to visualize and survey expansion of dedicated stent systems *in vitro*. So far, only few data about micro-CT are available, but micro-CT has already been described as an adequate tool for the assessment of single wires, lumen, endo-luminal plaques, cell sizes of stents [16], [36], [37], as well as for the assessment of complex stenting strategies on silicone bifurcated vessel models [38]. Importantly, this study provides data on autopsy heart samples from human subjects with CAD. Performing this investigation strictly in accordance with the guidelines of the local ethics committee, data were anonymized and no information about the CAD status was (and is) available. Nevertheless, in the real world clinical setting there is also often no information available on the CAD status and decisions regarding the interventional approach are made after the first angiographic projections. In the porcine heart samples no CAD was expected or detected. Therefore we do not think that the absence of information regarding CAD status will have significantly influenced our results. The findings of the present study are of practical interest in the context of the interventional treatment of coronary bifurcation lesions, the stent selection and the follow-up investigations in patients already having undergone Multi-Link Frontier stent implantation.

Meanwhile, new DSS with promising first-in-human experiences [39], [40] are available (Abbott Frontier™, Petal™, AST SLK-View™, BIGUARD™, Devax Axxess Plus™, Y-Med SideKick™, Stentys™, Capella Sideguard™, Invatec Twin-Rail™, Minvasys Nile Croco™, Tryton™ and TriReme Antares SAS™). The Asian Bifurcation Club designed a new DSS (BIGUIDE™) and initiated an ongoing phase 1 preclinical study [5]. The European Bifurcation Club outlined in a consensus report the importance of stent design, maximal stent cell size and final kissing balloon inflation [41]. The present study may give rise to further micro-CT investigations, yielding more data about the expansion behaviour of DSS and thereby contributing to the development of these devices.

### Limitations

Postmortem vessel wall resistance and the limited number of available autopsy heart samples (consequently limited number of investigated stents) might have influenced our data. Second, stent openings in real life are not always truly circular, so in these cases comparison with the data given by the manufacturer was made by assessing stent opening areas.

### Conclusions

Micro-CT is able to provide useful information for the evaluation and should be taken into account for the development of DSS. In real life, the proximal part and the stent length of the ML Frontier™ stent might show smaller expansion characteristics than in porcine heart models or water bath. Therefore, special consideration should be given to the stent deployment procedure of DSS, considering final IVUS or OCT to visualize (and if necessary optimize) stent expansion.
